# Mandibular distraction in neonates: indications, technique, results

**DOI:** 10.1186/1824-7288-38-7

**Published:** 2012-02-02

**Authors:** Enrico Sesenna, Alice S Magri, Cinzia Magnani, Bruno C Brevi, Marilena L Anghinoni

**Affiliations:** 1 Maxillo-Facial Surgery Division, Head and Neck Department (Head: Professor Enrico Sesenna); University and Hospital of Parma, Parma, Italy; 2 Neonatology Division, Department of Obstetrics, Gynecological and Perinatology Sciences; University and Hospital of Parma, Parma, Italy

## Abstract

**Background:**

The Pierre Robin Sequence features were first described by Robin in 1923 and include micrognathia, glossoptosis and respiratory distress with an incidence estimated as 1:8,500 to 1:20,000 newborns. Upper airway obstruction and feeding difficulties are the main concerns related to the pathology. Mandibular distraction should be considered a treatment option (when other treatments result inadequate).

**Patiants and methods:**

Ten patients between the ages of 1 month and 2 years with severe micrognathia and airway obstruction were treated with Mandibular Distraction Osteogenesis (MDO).

All patients underwent fibroscopic examination of the upper airway and a radiographic imaging and/or computed tomography scans to detect malformations and to confirm that the obstruction was caused by posterior tongue displacement. All patients were evaluated by a multidisciplinary team. Indications for surgery included frequent apneic episodes with severe desaturation (70%). Gavage therapy was employed in all patients since oral feeding was not possible. The two tracheotomy patients were 5 months and 2 years old respectively, and the distraction procedure was performed to remove the tracheotomy tube. All patients were treated with bilateral mandibular distraction: two cases with an external multivector distraction device, six cases with an internal non-resorbable device and two cases with an internal resorbable device. In one case, the patient with Goldenhar's Syndrome, the procedure was repeated.

**Results:**

The resolution of symptoms was obtained in all patients, and, when present, tracheotomy was removed without complications. Of the two patients with pre-existing tracheotomies, in the younger patient (5 months old) the tracheotomy was removed 7 days postoperatively. In the Goldenhar's syndrome case (2 years old) a Montgomery device was necessary for 6 months due to the presence of tracheotomy-inducted tracheomalacia. Patients were discharged when the endpoint was obtained: symptoms and signs of airway obstruction were resolved, PAS and maxillomandibular relationship improved, and tracheotomy, when present, removed. During the follow-up, no injury to the inferior alveolar nerve was noted and scarring was significant in only the two cases treated with external devices.

**Conclusion:**

Mandibular Distraction Osteogenesis is a good solution in solving respiratory distress when other procedures are failed in paediatric patients with severe micrognatia.

## Introduction

Infants with congenital craniofacial anomalies (CFAs) often display associated severe mandibular hypoplasia causing obstruction of the airway through retro-positioning of the tongue-base into the posterior pharyngeal airway [1].

Retro/micrognathia may be a feature of isolated Pierre Robin sequence (PRS), when PRS is not associated with other malformations (referred as non-syndromic PRS), or is part of several congenital craniofacial syndromes, such as Treacher Collins syndrome, Goldenhar syndrome, Nager syndrome, temporomandibular joint ankylosis and velocardiofacial syndrome [2-4].

The features of PRS were first described by Robin [5] and include micrognathia, glossoptosis and respiratory distress. Its incidence has been estimated as 1:8,500 to 1:20,000 newborns in the general population, with no gender predilection [4].

Robin [6] later revised the characteristics of the syndrome and included cleft palate as an additional factor that could be present. A wide range of clinical manifestations exists, but the main clinical problems faced by clinicians include upper airway obstruction and feeding difficulties [7].

It is clear that not every child with mandibular hypoplasia displays airway obstruction [8,9]. However, some patients may present an adequate airway when awake, but the obstruction may arise during feeding or sleeping when the pharyngeal muscle tone decreases. Thus, management regimes differ depending on the degree of upper airway obstruction (such as feeding difficulties, pulmonary infections).

Current treatments include non-surgical (prone positioning, nasopharyngeal tube/stenting, prolonged intubation), or surgical options (tongue-lip adhesion, tracheotomy, mandibular distraction osteogenesis). Recently, in neonates, mandibular distraction osteogenesis has been popularized in the literature and it is now widely accepted as the procedure of choice in the early management of airway obstruction due to craniofacial disproportion [4,10-13].

In this study, we report 10 selected paediatric patients with severe micrognathia and airway obstruction. The patients were treated by mandibular distraction osteogenesis when other non-surgical treatments failed to avoid tracheotomy or to remove the tracheotomy itself if present.

## Patients and methods

**Figure 1:**
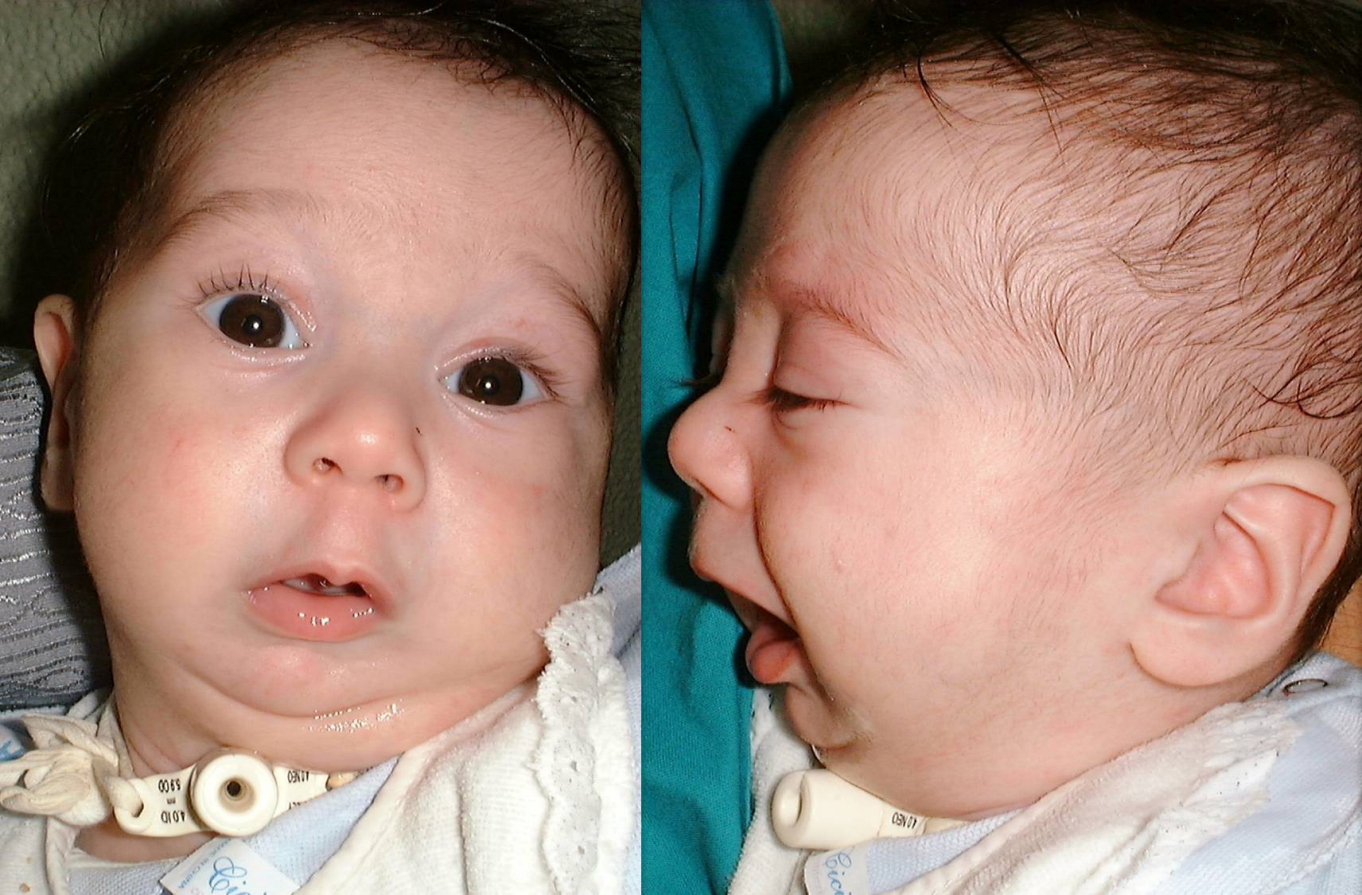
**Preoperative frontal and lateral views of an infant with Pierre Robin sequence**.

**Figure 2:**
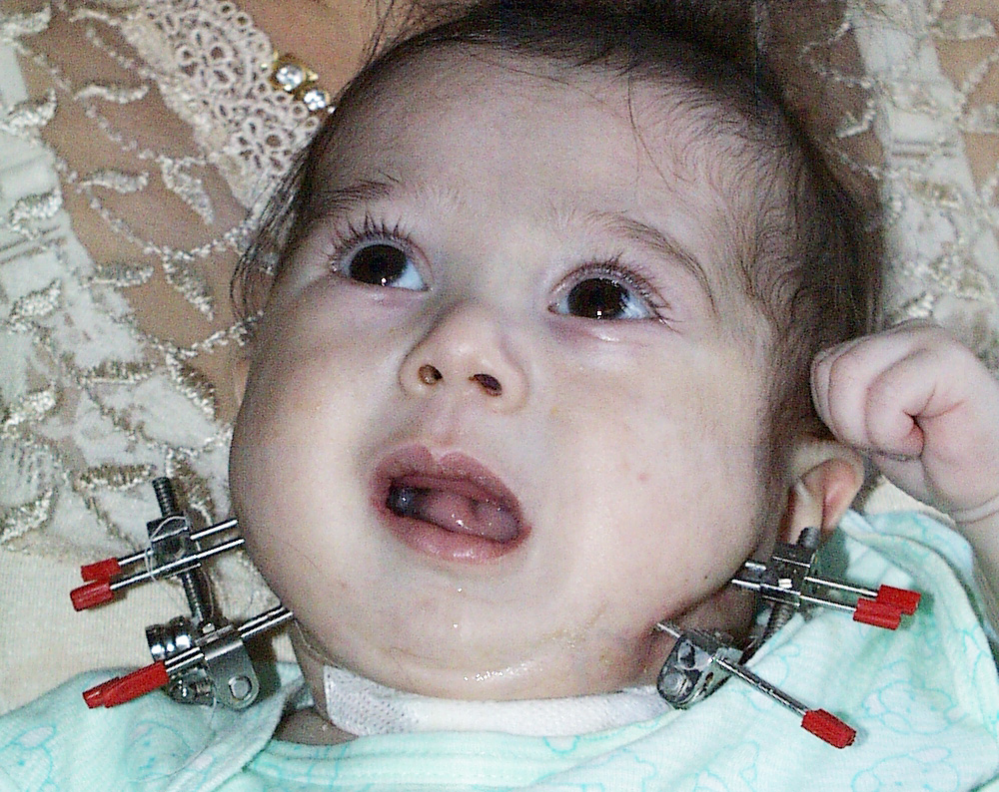
**Frontal view during distraction period with an extraoral distractor**.

**Figure 3:**
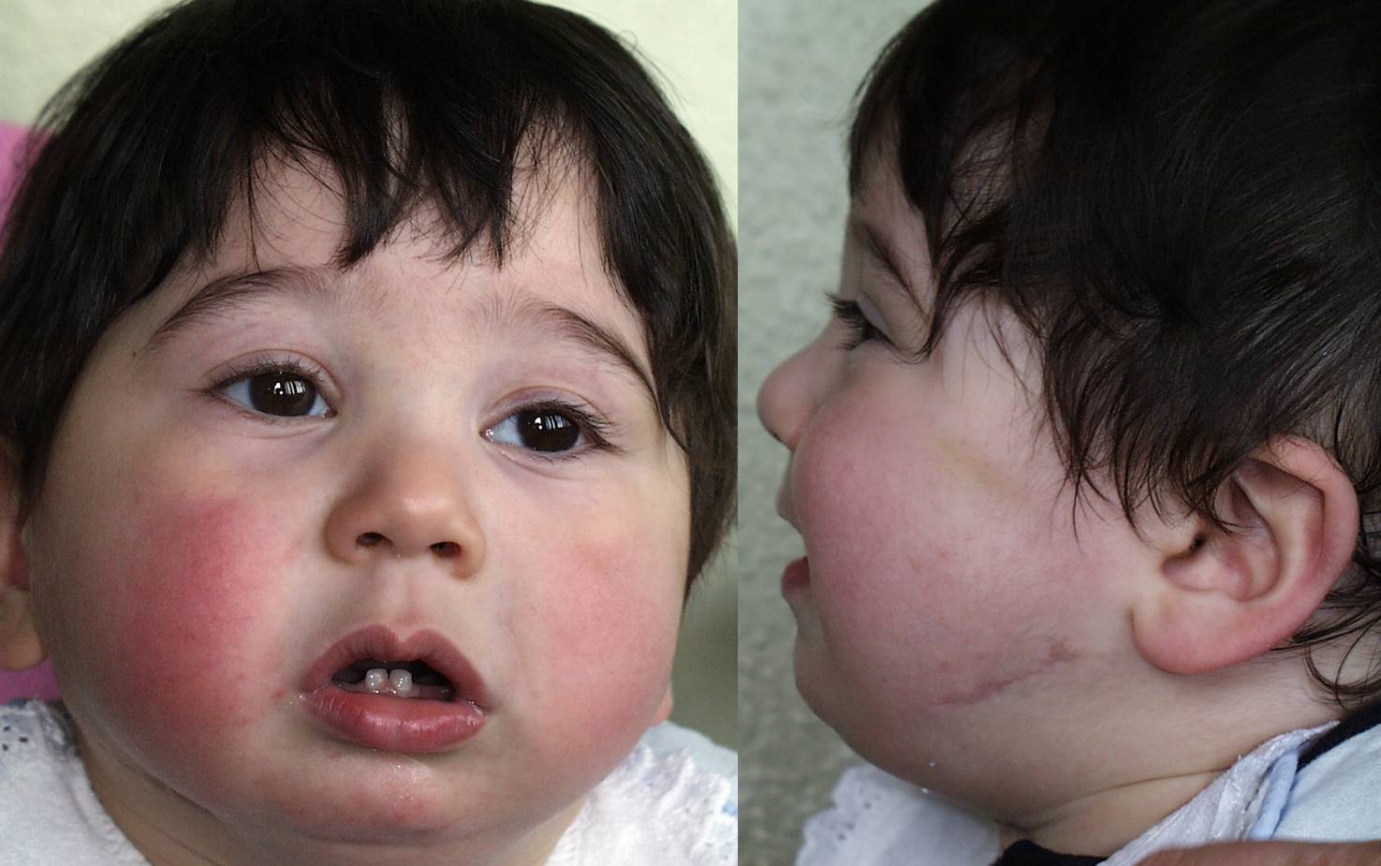
**Postoperative frontal and lateral views of the infant after mandibular distraction with external devices**.

In this study, we report our experience of 10 neonatal patients (9 with PRS, 1 with Goldenhar's syndrome; 7 males, 3 females) treated with bilateral mandibular distraction between January 2000 and December 2010 at the Parma University-Hospital, Department of Maxillo-Facial Surgery. Inclusion criteria were the presence of syndromic or non-syndromic mandibular hypoplasia, respiratory distress with episodes of severe desaturation (oxygen saturation below 70%; respiratory rate higher than 60/min), nasogastric tube feeding, lack of weight gain and the presence of a tracheostomy tube (2 cases). Exclusion criteria included central apnea, apnea that was dependent on other levels of airway impairment, such as laryngomalacia/tracheomalacia, and previous surgical procedures. All patients were submitted for: (1) fibroscopic examination of the upper respiratory system to detect malformations, such as tracheal stenosis, and to confirm that the obstruction was caused by posterior tongue displacement; (2) radiographic imaging and/or computed tomography scans to evaluate the posterior airway space (PAS), mandibular length, and maxillomandibular relationship (measurement between maxillary and mandibular central incisor buds or the anterior aspect of the maxillary and mandibular alveolar ridges). The mean age of the patients (3 cases with cleft palate) was 2.3 months (range, 1-5), except for one patient with Goldenhar's syndrome who was 2 years old. Two patients had previously undergone tracheotomy. All patients were evaluated by a team consisting in a geneticist, neonatologist, paediatric pneumologist, otolaryngologist, paediatric anaesthesiologist and a maxillofacial surgeon. Indications for surgery were repeated apneic episodes with severe desaturation (70%). Enteral nutrition was employed in all patients since oral feeding was not possible. In the Paediatric Intensive care unit, efforts were taken to avoid surgical treatment (prone positioning, tube feeding) leaving mandibular distraction as the last treatment option. The two tracheotomy patients were 5 months and 2 years old, respectively, and the distraction procedure was performed to remove the tracheotomy tube. All patients were treated with bilateral mandibular distraction: two cases with an external multivector distraction device (Figure 1, 2, 3), six cases with an internal non-resorbable device, and two cases with an internal resorbable device. In one case, (the patient with Goldenhar's syndrome), the procedure was repeated. The patients received general anaesthesia using either a pre-existing tracheotomy or oral intubation and preoperative prophylactic antibiotic therapy.

**Figure 4:**
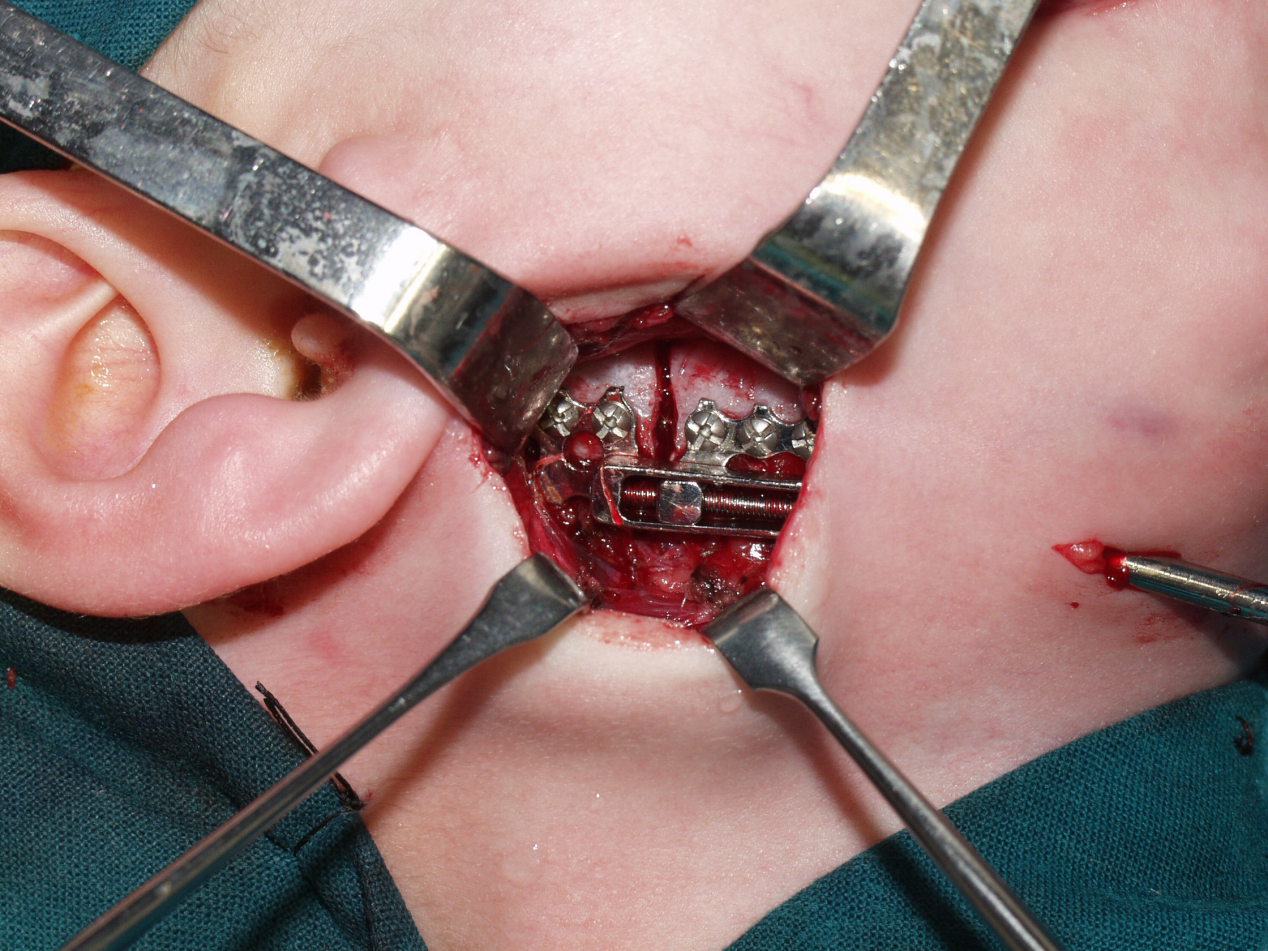
**Intraoperative view after placement of internal metal device**.

A surgical extra-oral incision was made 2 cm below the inferior mandibular margin, at the angle. A blunt dissection to reach the mandibular border and a subperiosteal dissection of the masseter muscle were performed. Osteotomy line was marked on the mandible outer cortex; the device was correctly positioned and fixed with four monocortical screws. The steps were repeated on the other side. The devices were then removed, a deepening of the osteotomy line with a fissure bur was produced and the osteotomy was completed, applying a rotational force with a chisel. Care was applied when opening the ostectomy site to preserve the alveolar nerve. The devices were re-fixed to the mandible using the same screw holes and assessed for stability (Figure 4). The skin was dissected anteriorly to the surgical incision, allowing the activation bar to merge from the skin. The devices were activated immediately (2 mm) with no latency period and the integrity of the inferior alveolar nerve assessed. The masseter muscle was repositioned and sutured to the internal pterigoid muscle with two resorbable sutures. In the following days, distraction continued 2 mm/day at each side for approximately 10 days (2 cm of lengthening) when the activation bar could be removed (if the device provided this option). The devices were removed, if not resorbable, following the consolidation period (2 months). We have described only the surgical technique of the internal device that is currently applied in our clinical practice. In the three initial cases, a preoperative tracheotomy was performed. In the final five cases, the patients were kept intubated in the Paediatric Intensive care unit until it was possible to safely remove the endotracheal tube. This typically occurred 6-7 days after device activation. Of the two patients with pre-existing tracheotomies, in the younger patient (5 months old) the tracheotomy was removed 7 days postoperatively. In the Goldenhar's syndrome case (2 years old) a Montgomery device was necessary for 6 months due to the presence of tracheotomy-inducted tracheomalacia.

**Figure 5:**
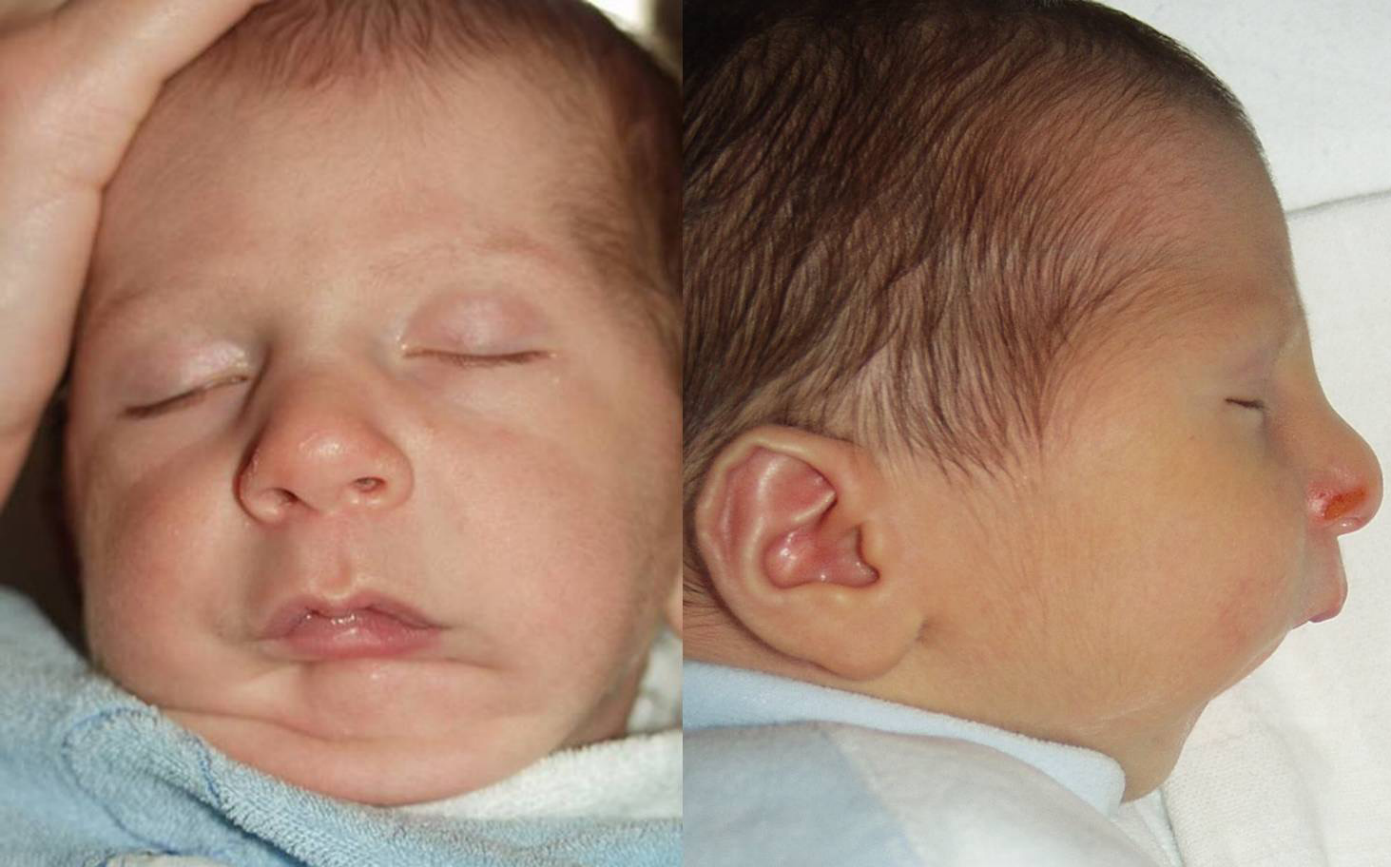
**Preoperative frontal and lateral views of an infant with Pierre Robin sequence**.

**Figure 6:**
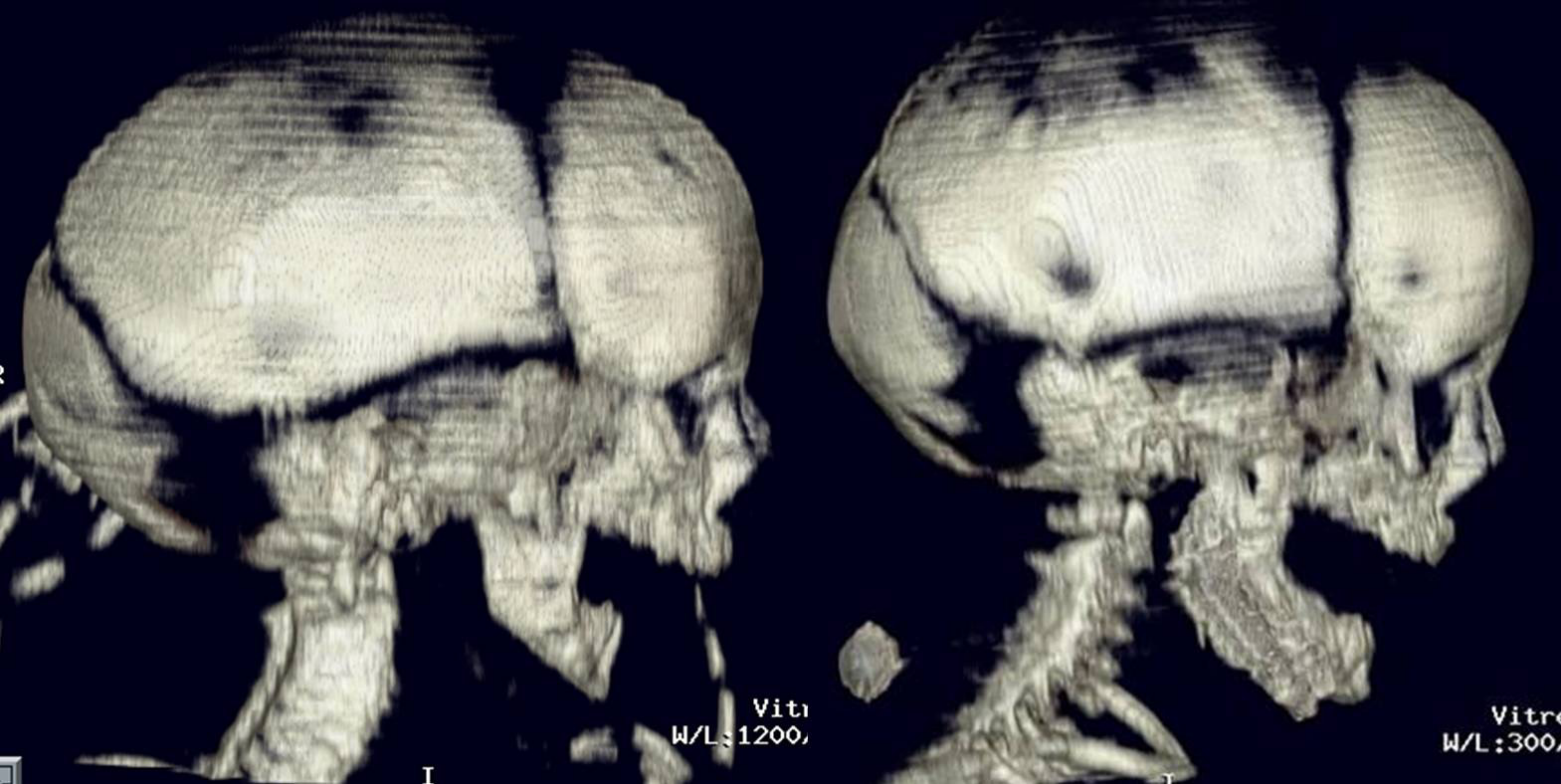
**Three-dimensional CT scan taken previously and during the distraction period**.

**Figure 7:**
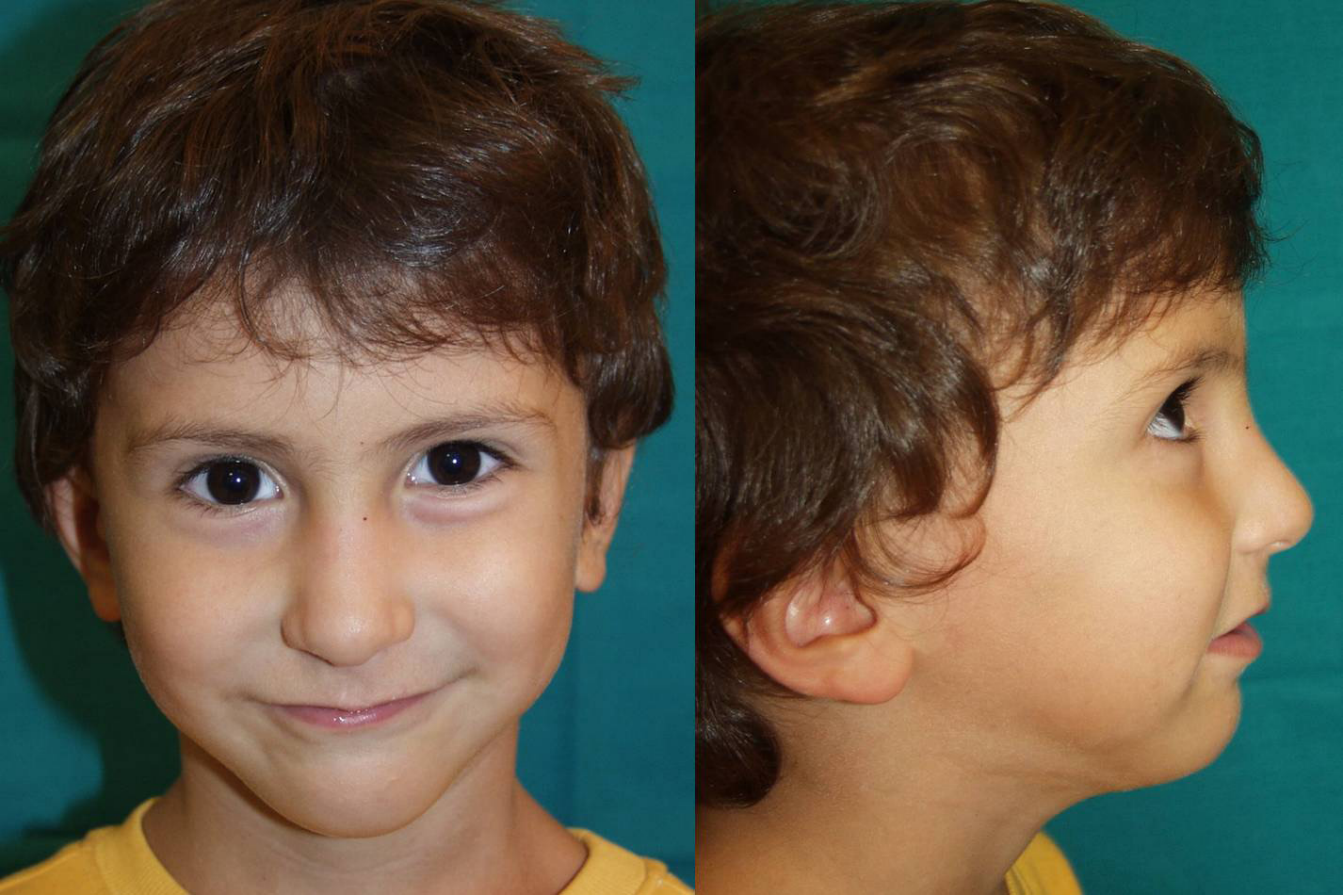
**Postoperative frontal and lateral views of the infant after mandibular distraction with internal device (note the inconspicuous scars)**.

Patients were discharged when the endpoint was obtained: symptoms and signs of airway obstruction solved, PAS and maxillomandibular relationship improved and tracheotomy, when present, removed. During the follow-up, no injury to the inferior alveolar nerve was noted and scarring was significant in only the two cases treated with external devices (Figure 5,6,7).

## Discussion and conclusions

Children with isolated PRS or associated with a range of other congenital malformations typically display a degree of airway obstruction [14]. The understanding of the pathogenesis of airway obstruction in PRS has rapidly evolved [15]. The dynamics of airway obstruction seems to be multi-factorial, with both anatomic and neuromuscular components. Anatomic abnormalities include retroposition of the mandible and reduced effectiveness of the genioglossus muscle in exerting anterior traction on the tongue. Delorme et al. [16] suggested that in such patients the genioglossus fibres are too short or too tight. They also noted that the tongue is not pushed back but is rotated posteriorly on its base. They proposed that the retruded mandible is the result, not the cause, of the tongue position.

Caouette-Laberge et. al. 1994 proposed the following clinical classification based on the severity of respiratory symptoms [17].

• Group I: adequate respiration in the prone position and bottle feeding.

• Group II: adequate respiration in the prone position but feeding difficulties requiring gavage.

• Group III: children with respiratory distress requiring respiratory support and gavage.

The clinical expression was heterogeneous: the severity of the airway complication varied from mild respiratory distress to extreme episodes of asphyxia.

Children with a mild degree of obstruction may present only subtle clinical signs, such as restless sleep, intermittent waking, and crying [18]. A lack of weight gain despite adequate nutritional intake is an additional indication of persistent airway obstruction. This is caused by the increased effort of breathing and increased calorie consumption in these children [4]. Severe airway distress is clinically manifested by persistent inspiratory stridor, several sternal and rib retractions, feeding difficulties (the necessity of tube feeding is frequent among these patients), and cyanosis that can result in cerebral hypoxia [18]. The priority in patients with severe micrognathia should be to maintain the free airway to avoid chronic obstruction that can lead to carbon dioxide retention, the development of pulmonary vasoconstriction, hypertension with right ventricular failure, and associated delayed development.

Micrognathia in the isolated PRS often improves or spontaneously resolves within the first 2 years of life, without surgical intervention for the mandible. This is possible because mandibular growth improves pharyngeal neuromuscular control and increases the airway space. This is clearly not valid in cases of syndromic infants with micrognathia who display an intrinsic anomaly in mandibular growth. From previous reports, it is evident that the management of syndromic children differs from isolated PRS [4]. Several authors have proposed an algorithm for the management of neonatal upper airway obstruction to avoid many of the unsuccessful outcomes [19,20]. According to these guidelines, we treated non-syndromic PRS patients with mandibular distraction when other treatments were inadequate.

In cases of mild upper airway obstruction, children can be managed by non-surgical treatment, including careful instruction regarding appropriate feeding techniques, an alertness to symptoms of increased obstruction, and lateral or prone positioning, where the infant is placed in ventral decubitus to displace the tongue anteriorly.

Nasopharyngeal intubation is recommended initially to alleviate the immediate hypoxia and present an adequate solution for a short time period but clinical evaluation is necessary to observe the adequacy of the procedure. The aim of this procedure is to maintain a good respiratory pattern by reducing the duration of tube feeding, promoting weight gain [21]. It is usually maintained for 7-10 days, when definitive treatment can be performed. When used as a definitive therapy it may be left in place for up to 8 weeks.

Patients with severe or refractory upper airway obstruction require more aggressive management. Surgical options most commonly include tongue-lip adhesion (TLA), tracheotomy, and mandibular distraction osteogenesis (MDO).

The tongue-lip adhesion procedure was introduced by Shukowsky in 1911 [22] and popularized by Douglas in 1946 [23]. It was designed to alleviate upper airway obstruction by correcting abnormal tongue positioning. The procedure is clearly not physiological, because it prevents the normal tongue movements and reported complications include dehiscence, tongue lacerations, injuries to the Wharton's ducts, wound infection, scar deformation and pneumonia "*ab ingestis*" [2,14,24].

Mandibular traction devices have also been used: Stellmach [25] reported the use of two circummandibular wires in the symphyseal area, attached to a 70-g weight, with an upwards traction. The use of circummandibular wires attached to a skull cap with an "outrigger" bar as an adjunct to glossopexy has also been reported. These treatment modalities can be effective, but must be performed for a long time period (2-3 months) and require hospitalization [2].

Others procedures that have been described include transfixion of the tongue by a transmandibular Kirschner wire placed anterior to the mandibular angle, as proposed by Hadley 1963 [26], but problems with this procedure include instability and impracticability. Hyomandibulopexy (by Bergoin 1971 [27] and by Lapidot 1976 [28]) is designed to anchor the hyoid bone to the mandible anteriorly and to bring the base of the tongue forward. Interference with mandibular growth is considered a drawback of this procedure. Subperiosteal release of the floor of the mouth musculature was described by Delorme [16] with the concept that the musculature of the floor of the mouth when under increased tension pushes the tongue upwards and backwards. Early release of this musculature should allow the tongue to return to a more normal position. However, there are no published studies with objective measurements that demonstrate the benefits of this technique [1]. In 1998, Myer [14] reported the use of tracheotomy regarding the issue of long-term airway management. Tracheotomy in neonatal airway obstruction may be life-saving but is associated with complications and developmental problems. This procedure can also be associated with severe complications, such as accidental decannulation and obstruction of the tube, which can occur at any time during the entire period of the tracheotomy. Early complications include haemorrhage from stomal varices, pneumothorax, pneumomediastinum, and minor complications include tracheitis, pneumonia, tracheal granulations, respiratory infections, subglottic stenosis, and cricoid cartilage injury [4,29]. Once in place, the tracheotomy is usually necessary for 1-4 years. During the time of cannulation, home care remains a significant burden for the family and medical care systems. Additionally, typically, skilled home care for a child with a tracheostomy is difficult to obtain.

The average age at decanulation is 3.1 years and long-term problems, such as growth retardation, delayed speech, articulation difficulties, and behavioural problems [30] are present in up to 50-75% of cases [31]. The most frequent complication is tracheomalacia.

Recently, MDO has emerged as an alternative method for relieving airway obstruction in paediatric patients with severe mandibular hypoplasia to avoid tracheotomy and improve oral feeding. Distraction osteogenesis is a surgical orthopaedic technique that was first introduced to lengthen the long bones of the body [32]. This technique is based on the principle that tension stimulates histogenesis with bone formation (Codivilla of Bologna, Italy, described the technique as early as 1905) [33].

McCarthy et al. applied these concepts to treat underdeveloped mandibles in the early 1990s [34]. From that time, numerous reports have been published, and the technique has evolved and been applied throughout the craniofacial skeleton, including the maxilla, midface, orbits, and cranium.

Mandibular lengthening obtained with MDO provides an alternative to traditional methods of airway management in infants with micrognathia. As the mandible is lengthened, the tongue base moves forward by its anterior muscular attachments to the mandible, increasing the airway space and relieving airway obstruction.

This procedure has been found to be safe and the surgical scars cosmetically acceptable. Some complications include damage to the inferior alveolar nerves, infections, failure of distraction, dislodgement of pins or distractors and damage to the tooth buds.

Distraction osteogenesis consists of four primary and consistent phases: (1) device placement and osteotomy, (2) a latency period of primary healing (not in these patients), (3) active distraction (at a rate of 2 mm/d until the desired level of distraction is achieved). Patients are given 5 days of antibiotics, and are restricted to a soft diet. At the end of the procedure (4), after completing the distraction, the devices are left in place for an additional 4-6 weeks to allow the regenerated bone to consolidate [35].

Three types of device exist: external, internal and resorbable, and uni/multivectorial. External devices are easier to apply but cause scarring due to the pins. Other problems such as pin site infections and the dislodgement of devices has been observed. External devices can be removed with local aesthesia but frequently require revision of the scar caused by the pin moving through the skin (Figure 3).

Internal devices are placed directly on the mandibular bone with improved compliance by the patients and parents. When the end point of active distraction is reached, some internal devices offer the possibility to remove the activation bar that emerges from the skin during the activation phase.

**Figure 8:**
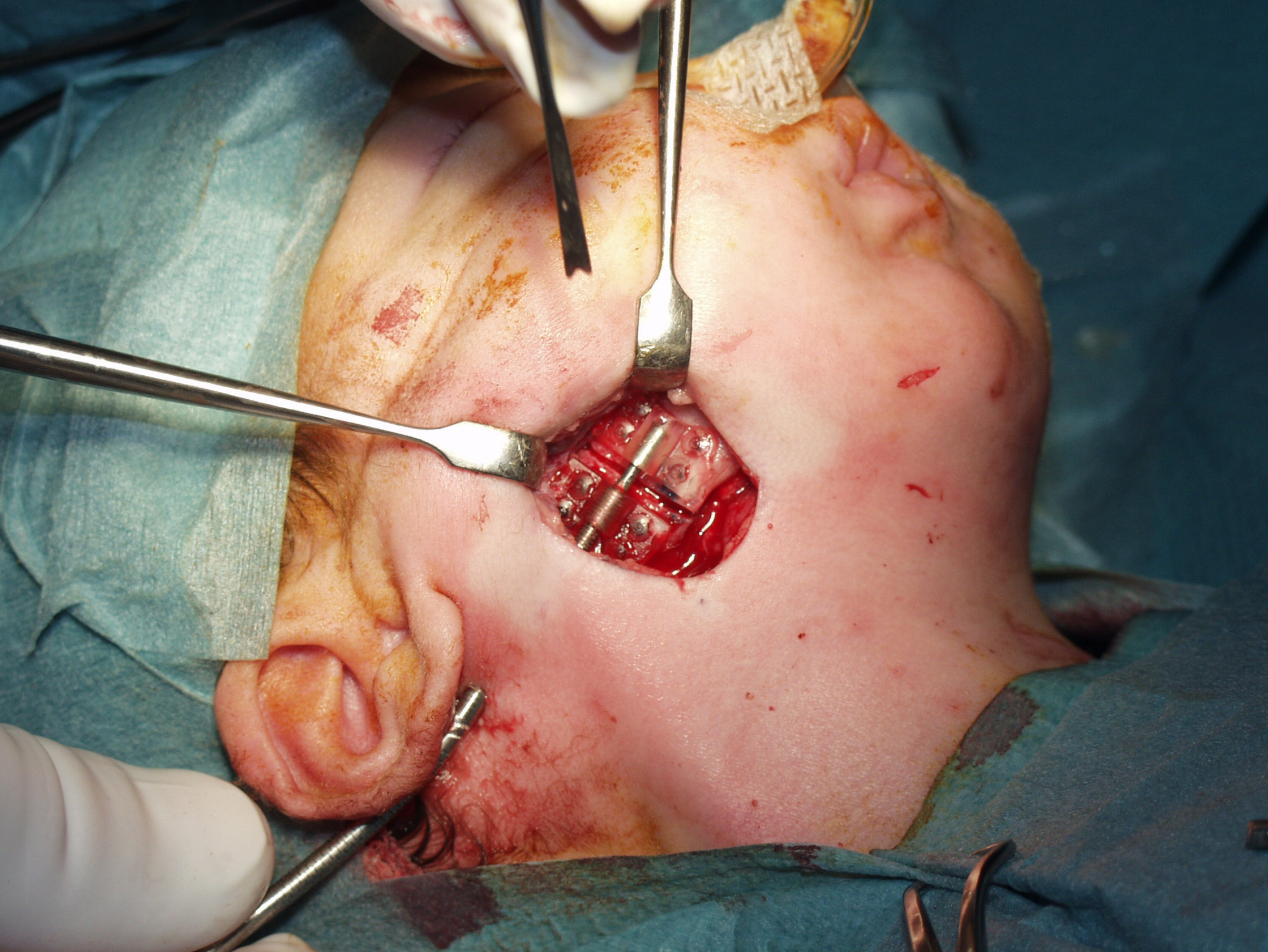
**Intraoperative view: mandibular resorbable distraction device in place**.

Following the consolidation period, the non-resorbable devices must be removed with a second surgical procedure under general aesthesia. This is the major shortcoming of this kind of technique. Recently, internal resorbable devices have been proposed that can be left in place as they resorb during a 6-8 month period, avoiding a second surgical procedure (Figure 8). This procedure improves comfort and reduces the risk of infection during consolidation. In our experience, we began with the external devices (2 cases) [36], as these were the only ones available. We then moved to the internal non-resorbable distractors (6 cases). In the last two cases internal resorbable distractors were used.

## Consent

Written informed consent was obtained from the patient for publication of this article and accompanying images.

## List of abbreviations

CFAs: congenital craniofacial anomalies; MDO: Mandibular Distraction Osteogenesis; PRS: Pierre Robin sequence; PAS: posterior airway space; TLA: tongue-lip adhesion.

## Competing interests

The authors declare that they have no competing interests.

## Authors' contributions

ES performed surgery and participated in the sequence alignment and drafted the manuscript, ASM participated in the sequence alignment and drafted the manuscript, CM carried out the molecular genetic studies, BCB performed surgery and participated in the sequence alignment, MLA participated in the sequence alignment. All authors read and approved the final manuscript.
